# HMGB3 is Associated With an Unfavorable Prognosis of Neuroblastoma and Promotes Tumor Progression by Mediating TPX2

**DOI:** 10.3389/fcell.2021.769547

**Published:** 2021-12-20

**Authors:** Xiaodan Zhong, Songling Zhang, Yutong Zhang, Zongmiao Jiang, Yanan Li, Jian Chang, Junqi Niu, Ying Shi

**Affiliations:** ^1^ Department of Pediatric Oncology, The First Hospital of Jilin University, Changchun, China; ^2^ Department of Obstetrics and Gynecology, The First Hospital of Jilin University, Changchun, China; ^3^ Department of Endocrinology and Metabolism, The First Hospital of Jilin University, Changchun, China; ^4^ Department of Pediatrics, The First Hospital of Jilin University, Changchun, China; ^5^ Department of Hepatology, The First Hospital of Jilin University, Changchun, China

**Keywords:** neuroblastoma, HMGB3, proliferation, cell cycle, TPX2

## Abstract

Neuroblastoma (NB) is the most common solid tumor apart from central nervous system malignancies in children aged 0–14 years, and the outcomes of high-risk patients are dismal. High mobility group box 3 (HMGB3) plays an oncogenic role in many cancers; however, its biological role in NB is still unclear. Using data mining, we found that HMGB3 expression was markedly elevated in NB patients with unfavorable prognoses. When HMGB3 expression in NB cell lines was inhibited, cell proliferation, migration, and invasion were suppressed, and HMGB3 knockdown inhibited NB tumor development in mice. RT−PCR was employed to detect mRNA expression of nine coexpressed genes in response to HMGB3 knockdown, and TPX2 was identified. Furthermore, overexpression of TPX2 reversed the cell proliferation effect of HMGB3 silencing. Multivariate Cox regression analysis indicated that HMGB3 and TPX2 might be independent prognostic factors for overall survival and event-free survival, which showed the highest significance (*p* < 0.001). According to the nomogram predictor constructed, the integration of gene expression and clinicopathological features exhibited better prognostic prediction power. Furthermore, the random forest algorithm and receiver operating characteristic curves also showed that HMGB3 and TPX2 played important roles in discriminating the vital status (alive/dead) of patients in the NB datasets. Our informatics analysis and biological experiments suggested that HMGB3 is correlated with the unfavorable clinical outcomes of NB, and plays an important role in promoting cell growth, proliferation, and invasion in NB, potentially representing a new therapeutic target for tumor progression.

## Introduction

Neuroblastoma (NB) is the third most common cancer in children under the age of 15 years, and originates from neural crest-derived sympathetic adrenal precursors, accounting for approximately 7% of pediatric malignancies; however, it is responsible for nearly 15% of childhood cancer mortality (Ward et al., 2014; Mullassery and Losty, 2016; Zafar et al., 2021). Over the past 30 years, multimodality treatment strategies have been developed all over the world; nonetheless, the outcomes of high-risk patients remain dismal (less than 50%), and one-half of high-risk NB patients are confronted with refractory disease, progression, and even death (Pinto et al., 2015; Bosse and Maris, 2016; Ahmed et al., 2017).

A study enrolling 240 cases reported a low mutation frequency in NB, less than 20% in total (Pugh et al., 2013). Such relatively uncommon somatic mutations in NB have made it challenging for existing treatment strategies to target frequently mutated oncogenic driver genes. On the other hand, the application of diverse oncogene-targeting drugs, such as CDK4/6 inhibitors (Rihani et al., 2015; Geoerger et al., 2017), AURKA inhibitors (DuBois et al., 2016), and ALK inhibitors (Whittle et al., 2017), has brought some hope to high-risk/refractory/relapsed patients. Nonetheless, it is still far from sufficient, and novel therapeutic targets are urgently needed.

High mobility group box 3 (HMGB3) belongs to the high mobility group protein subfamily, which also includes HMGB1, HMGB2, and HMGB4 (Reeves, 2015). HMG box family members play important roles in cancer by binding to DNA structure and multiple other patterns (Niu et al., 2020). In particular, HMGB1 plays paradoxical roles in promoting cancer cell proliferation and inhibiting malignant cell survival (Kang et al., 2013). HMGB1 exerts dual functions in and out of cancer cells via multiple signaling pathways, such as immunity, autophagy, and inflammation. Moreover, HMGB1 is an important paralog of HMGB3. Over the past decade, the carcinogenic effects of HMGB3 have been reported in a variety of tumors, including colorectal cancer (CRC) (Zhang et al., 2017), breast cancer (BC) (Gu et al., 2019), cervical cancer (Li Z. et al., 2020; Zhuang et al., 2020), and non-small cell lung cancer (NSCLC) (Li Y. et al., 2020). Additionally, HMGB3 depletion is suggested to reduce the cisplatin resistance of ovarian cancer cells (Mukherjee et al., 2019). However, the expression and function of HMGB3 in NB remain unknown.

## Materials and Methods

### Bioinformatics Analysis

To analyze the mRNA expression of HMGB3 in NB, the GSE49710, GSE16476, and GSE120572 datasets were downloaded from the Gene Expression Omnibus (GEO) database (https://www.ncbi.nlm.nih.gov/geo/), whereas survival information and TARGET-249 data were downloaded from the R2 database (https://r2.amc.nl).

### Cell Culture

The NB cell lines SK-N-SH, SH-SY5Y, and SK-N-BE 2) were cultured in MEM supplemented with 10% fetal bovine serum (FBS) and 1:100 penicillin-streptomycin solution. SK-N-AS cells were cultured in DMEM containing 10% FBS. All cell lines were purchased from Procell Life Science & Technology Co., Ltd. (Wuhan, China), and were verified by short tandem repeat profiling. The 3D culture was conducted in shRNA and sh-NC cells. In brief, 1 × 10^5 cells/ml were cultured in MEM/DMEM supplemented with 10% FBS, and 20 µl of cell culture was added onto the lid (inside) of 60 × 15 mm cell culture dishes. Thereafter, the lid was flipped, and the cells were cultured for 5 days. Afterward, the lid was flipped again, and images were captured.

### RNA Extraction and Real-Time Reverse Transcription PCR

After lentivirus transfection of sh-HMGB3 or sh-NC SK-N-SH and SK-N-AS cells for 48 h, total RNA was extracted from NB cells using the TRAN Easy Pure RNA kit. Then, cDNA was obtained using the cDNA Synthesis SuperMix kit, and the gene expression of HMGB3 and TPX2 in NB cells was examined by qRT−PCR performed using the SYBR Green mix kit. β-actin mRNA (ACTB) was used as the endogenous reference. All primers used in this study were purchased from Sangon Biotech Co., Ltd. (Shanghai, China). The primer sequences are listed in Table 1. The mRNA expression of all genes was calculated using the 2^–ΔΔct^ method (Livak and Schmittgen, 2001).

**TABLE 1:** Primer sequences used for qPCR.

Gene	Forward	Reverse
HMGB3	CCA​AGA​AGT​GCT​CTG​AGA​GGT​G	CTT​CTT​GCC​TCC​CTT​AGC​TGG​T
TPX2	TTC​AAG​GCT​CGT​CCA​AAC​ACC​G	GCT​CTC​TTC​TCA​GTA​GCC​AGC​T
CCNB2	CAA​CCA​GAG​CAG​CAC​AAG​TAG​C	GGA​GCC​AAC​TTT​TCC​ATC​TGT​AC
CDCA2	GAG​GCA​GGA​AAA​GAG​TCC​GAG​A	CTC​CGA​CGT​TTG​GAG​GAC​AAC​A
MCM10	TCA​AGG​AAC​TGA​TGG​ACC​TGC​C	CTC​CAA​CAT​CCG​CTG​CTT​CTG​T
BUB1B	GTG​GAA​GAG​ACT​GCA​CAA​CAG​C	TCA​GAC​GCT​TGC​TGA​TGG​CTC​T
NCAPH	CCT​CAA​TGT​CTC​CGA​AGC​AGA​TC	TGT​AGT​CCT​GGC​AGT​GGA​GAG​T
CENPE	GGA​GAA​AGA​TGA​CCT​ACA​GAG​GC	AGT​TCC​TCT​TCA​GTT​TCC​AGG​TG
RNASEH2A	GCC​GTG​AAG​AAA​TGG​CAG​TTC​G	GTG​CTC​CTT​CAA​CCA​CGC​TTT​TG
GINS2	AGC​CAA​ACT​CCG​AGT​GTC​TGC​T	CTT​GTG​TGA​GGA​AAG​TCC​CGC​T
ACTB	CAC​CAT​TGG​CAA​TGA​GCG​GTT​C	AGG​TCT​TTG​CGG​ATG​TCC​ACG​T

### Lentivirus Transfection

To achieve HMGB3 silencing, three candidate shRNAs and sh-NC were designed by GenePharma (Shanghai, China). Cells were transfected with recombinant lentiviral transduction particles with green fluorescent protein (GFP). Afterward, lentiviral fluid was added to the cells cultured in MEM and incubated for 6 h, and the medium was replaced with fresh MEM containing 10% FBS. The transfection efficiency was evaluated under a fluorescence microscope after 48 h. The sh-HMGB3 sequences are listed in Table 2.

**TABLE 2:** Sequence of sh-HMGB3.

	Sequence 5′-3′
shRNA1	5′-GGG​CAA​GAT​GTC​CGC​TTA​TGC-3′
shRNA2	5′-GGA​AGA​CGA​TGT​CCG​GGA​AAG-3′
shRNA3	5′-GGA​AAG​TTT​GAT​GGT​GCA​AAG-3′
shRNA-NC	Empty vector

### Immunoblot Analysis

NB cells were transfected with sh-HMGB3/sh-NC or TPX2-OE lentivirus for 72 h, and then total protein was extracted from cell lysates using RIPA solution, mixed with 5× protein loading buffer, incubated at 100°C for 10 min, and then separated by 10–12% SDS−PAGE. After electrophoresis, the proteins were transferred onto nitrocellulose membranes; then, the membranes were blocked with PBS containing 5% bovine serum albumin (BSA) for 1 h at room temperature. Thereafter, the membranes were incubated with indicated primary antibodies (HMGB3 and TPX2, 1:2000; Boster, United States) for 2 h at room temperature, washed with PBST (0.5% Tween-20) three times and stored at 4°C overnight. Subsequently, the membranes were further incubated with alkaline phosphatase-conjugated goat-anti-rabbit antibodies for 1 h at room temperature, washed three times, and detected using the BCIP/NBT alkaline phosphatase color development kit. The protein band densities were quantified using ImageJ 1.8.0 software (National Institutes of Health).

### CCK8 Assay

To demonstrate cell growth, we conducted a cell counting-kit 8 (CCK-8) assay to detect viable cells. Briefly, sh-HMGB3 and sh-NC cells were resuspended in MEM/DMEM containing 10% FBS to a final concentration of 1 × 10^4 cells/ml. Later, 100 µl suspension was added into 96-well plate, and then 10 µl CCK-8 reagent was also added and incubated for 2 h in an incubator at 37°C and 5% CO_2_. Finally, the absorbance was measured at 450 nm for 8 consecutive days.

### Colony Formation Assay

A total of 1000 sh-HMGB3/sh-NC cells/well were seeded into 6-well plates and incubated for 10 days in an incubator at 37°C and 5% CO_2_. Thereafter, the clones were fixed in paraformaldehyde for 30 min, stained with crystal violet solution (Beyotime, China) for 20 min, and washed with water three times. Finally, the clones were imaged and counted. Clone formation rate (%) = (number of clones/number of seeded cells) × 100%.

### Wound Healing Assay

To examine cell migration, a wound healing assay was performed. In brief, SK-N-SH, and SK-N-AS cells were seeded in MEM/DMEM at a density of 2 × 10^5 cells/well into the 12-well plates, and HMGB3 expression was silenced as described before. After overnight culture, a 200-μl tip was used to make a scratch in the cell monolayer, and wound closure was observed for 8 h. For each well, two images in the same area were taken and analyzed by ImageJ software. Wound healing rate (%) = [(Area at 0 h)-(Area at 8 h)]/(Area at 0 h) × 100%.

### Transwell Assay

Precooled serum-free DMEM/MEM was used to dilute the Matrigel matrix (Corning, United States) to a concentration of 400 μg/ml; thereafter, 100 µl of the diluted solution was coated onto the upper Transwell culture chamber and incubated overnight at 37°C and 5% CO_2_. Subsequently, the cells were resuspended in serum-free DMEM/MEM to a concentration of 2 × 10^5 cells/ml, and 100 μl cell suspension was added to the upper chamber coated with Matrigel, while 600 µl of MEM/DMEM supplemented with 20% FBS was added to the lower chamber. After 40 h, cells on the upper chamber surface were removed and fixed with 4% paraformaldehyde for 30 min. Later, the chamber was stained with 1% crystal violet for 20 min, and images were taken from five fields of view using an inverted microscope. The number of cells crossing the bottom chamber was quantified.

### Tumor Xenograft Assay

Animal experiments were approved by the Laboratory Animal Ethics Committee of the First Hospital of Jilin University, and the animals were cared for in agreement with institutional ethics guidelines. Ten BALB/c nude female mice (6 weeks old) were anesthetized and subcutaneously injected with 2 × 10^6 sh-NC or sh-HMGB3 SK-N-SH cells into the right flank. When the tumor diameters of the mice reached 15 mm, all mice were sacrificed. The weight and volume of the tumor were weighed and calculated, and tumor volume = (width)^2^×length/2.

### Immunocytochemistry

NB cells were seeded into 12-well plates and incubated in an incubator at 37°C and 5% CO_2_ overnight. Thereafter, the cells were fixed in 4% paraformaldehyde for 5 min, and 0.5% Triton-X-100 was added to increase the cellular membrane permeability. Next, the cells were blocked with 5% BSA for 30 min, incubated with Ki-67 antibody (1:1,000) at 37°Cfor 1 h, incubated with HRP-conjugated goat anti-rabbit IgG polymer for 30 min, and stained with DAB and hematoxylin. After washing, images were acquired under an inverted microscope, and the positive cell rate was calculated.

### Correlation Analysis of HMGB3 and the Coexpressed Genes

We calculated the Pearson correlation coefficient (PCC) of HMGB3 and other genes in four NB datasets, GSE49710, GSE16476, GSE120572, and TARGET-249. Then, we selected the genes with PCC >0.7, which were considered coexpressed genes, for further experiments.

### Methods for Estimating the Importance of HMGB3 and TPX2 in NB Prognosis

The random forest algorithm (Carolin Strobl et al., 2007) is an important and excellent feature selection approach among machine learning algorithms, that can rank the importance of diverse features. In this study, the random forest algorithm was utilized to estimate the importance of HMGB3, TPX2, and other clinicopathological characteristics for the vital status of NB patients. Meanwhile, mean decrease accuracy (MDA) and mean decrease Gini (MDG) were employed as parameters to estimate the importance. In addition, the receiver operating characteristic (ROC) curve was plotted to test the discriminating abilities of the two genes and other risk factors in vital status, and the area under the curve (AUC) values were used for comparison.

### Statistical Analysis

Statistical analysis was conducted using Graphpad Prism 8.0 and R 3.6.2 software. Univariate and multivariate Cox regression analyses were performed using the R package “survival,” and Harrell’s concordance index (C-index) was acquired to assess the model performance. Measurement data are expressed as the mean ± standard deviation (SD). Differences between two groups were compared by Student’s t-test. The relative gene expression level was log_2_ transformed. Differences in gene expression between two groups were compared using the Mann−Whitney *U* test. The relationship of gene expression with clinicopathological features was analyzed using the nonparametric χ^2^ test. Survival analysis was conducted using the log-rank test and visualized by the Kaplan−Meier plot. ggforest plots were generated using the R package “survminer”. The nomogram plot was generated using the R package “rms”. To test the predictive performance of diverse features, the AUC values of the ROC curve were calculated and plotted using the R package “pROC”. Moreover, the random forest algorithm was conducted using the R package “randomForest”. All statistical tests were two-sided and a difference of *p* < 0.05 was considered statistically significant. All experiments were independently conducted in triplicate.

## Results

### HMGB3 Exhibits Abnormally High Expression in NB Patients With Unfavorable Prognoses

**FIGURE 1:**
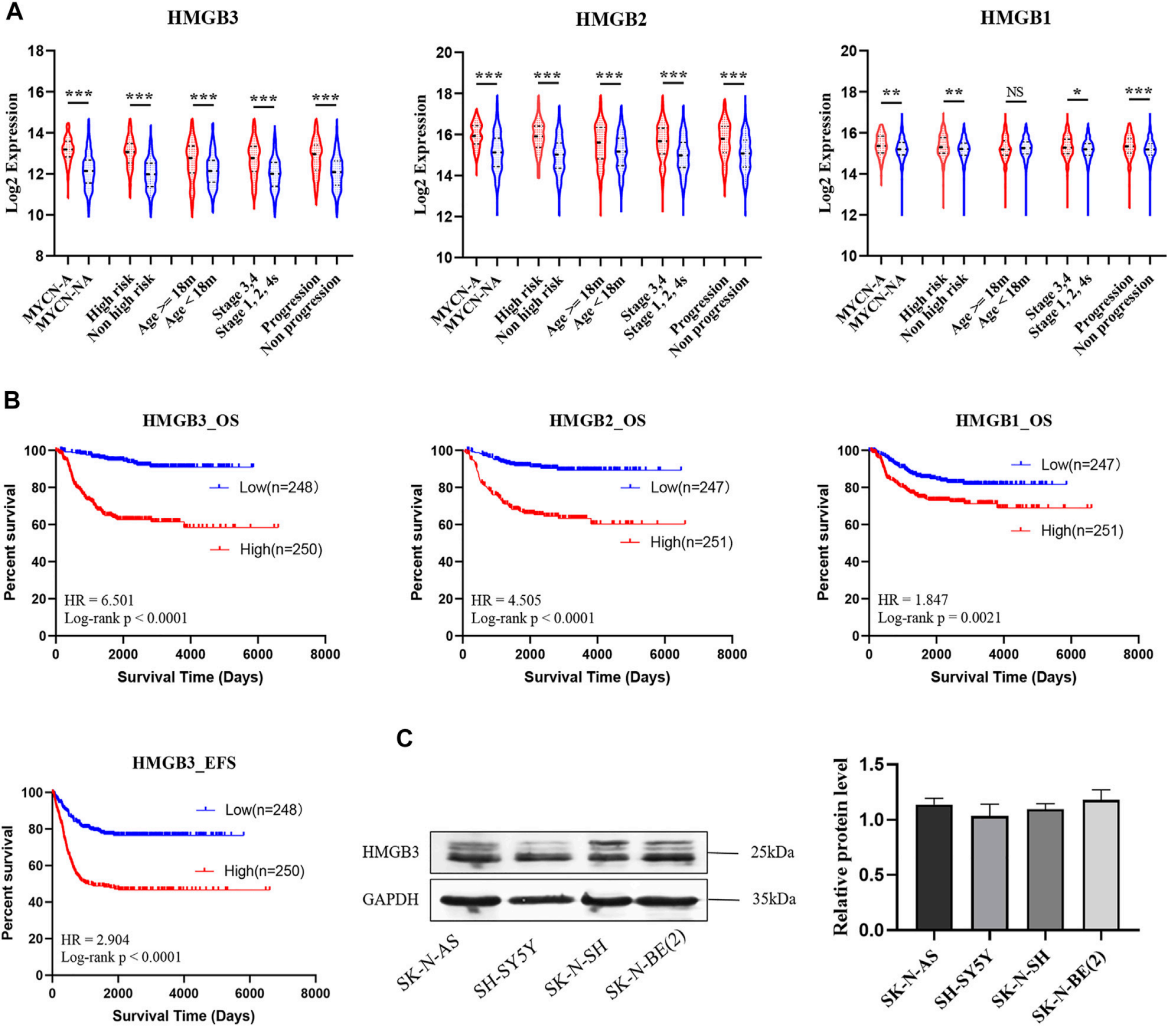
HMGB3 is abnormal highly expressed in human neuroblastoma with unfavorable prognoses in GSE49710. **(A)** HMGB3 over-expressed in unfavorable prognostic groups of NB patients. **(B)** High expression of HMGB3 correlated to inferior overall survival. **(C)** Expression of HMGB3 in four NB cell lines. *p < 0.05, **p < 0.01, and ***p < 0.001.

HMGB family members (HMGB1, HMGB2, and HMGB3) were highly expressed in NB patients with unfavorable prognosis in GSE49710, and the difference in HMGB3 expression was the most significant (Figure 1A). High expression of HMGB3 was correlated with inferior overall survival (OS) and event-free survival (EFS) (Figure 1B). Furthermore, we analyzed the correlations between HMGB expression and clinicopathological features among 493 NB patients. HMGB2/3 levels were correlated with the following features: age≥18 months, MYCN amplification, high risk, advanced stage, progression, and death (*p* < 0.0001, Table 3). Moreover, the relationship between HMGB3 expression and clinicopathological features in three other NB datasets (GSE16476, TARGET-249, and GSE120572) was analyzed, and similar results were obtained (Supplementary Tables S1–S3). Based on the above results, we further examined HMGB3 expression levels in the four NB cell lines SK-N-SH, SK-N-AS, SH-SY5Y, and SK-N-BE 2) by western blotting (WB) (Figure 1C). The results suggested that expression of HMGB3 in all 4 cell lines was similarly high. SK-N-SH and SK-N-AS cell lines were chosen for further analysis.

**TABLE 3:** The relationship between HMGBs expression and clinicopathological features in NB patients.

Features	HMGB1			HMGB2			HMGB3		
	High	Low	*p* value	High	Low	*p* value	High	Low	*p* value
Gender									
Male	129	155	0.015	133	151	0.979	148	136	0.398
Female	119	90		99	110		100	109	
Age									
≥18 m	90	101	0.302	115	76	0.001	123	68	1.04E-06
<18 m	158	144		135	167		125	177	
MYCN									
Amplified	59	33	0.005	79	13	1.79E-13	85	7	<2.2E-16
Non-Amp	189	212		171	230		163	238	
High risk									
Yes	98	77	0.075	139	36	<2.2E-16	142	33	<2.2E-16
No	150	168		111	207		106	212	
Stage									
3, 4	132	111	0.095	163	80	1.48E-12	162	81	1.51E-12
1, 2, 4s	116	134		87	163		86	164	
Progression									
Yes	106	74	0.005	124	56	1.65E-09	125	55	2.12E-10
No	142	171		126	187		123	190	
Death									
Yes	66	38	0.004	83	21	4.98E-11	87	17	4.45E-14
No	182	207		167	222		161	228	

### Silencing HMGB3 Inhibits Cell Proliferation, Migration, and Invasion *in Vitro* and *in Vivo*

**FIGURE 2:**
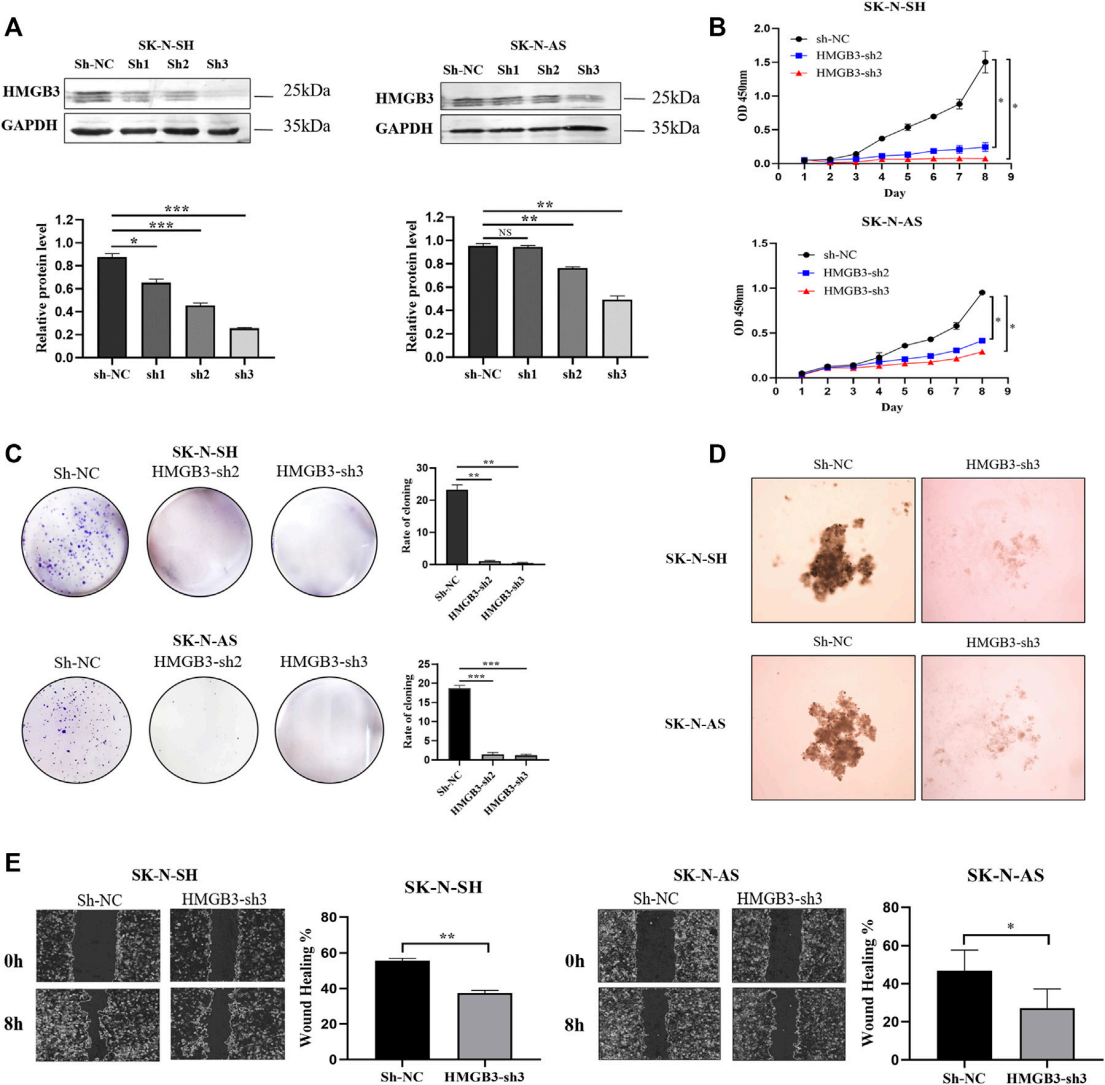
Knockdown of HMGB3 inhibited cell proliferation and migration in NB cells *in vitro*. **(A)** Protein expression of HMGB3 after lentivirus transfection (sh-NC, sh-RNA1, sh-RNA2, and sh-RNA3). **(B–E)** CCK8, Colony formation, 3D cell culture, and wound healing assay for SK-N-SH and SK-N-AS cells with/without HMGB3 knockdown. *p < 0.05, **p < 0.01, and ***p < 0.001.

To determine the biological function of HMGB3 in NB cells, lentivirus with sh-HMGB3 or empty vector sh-NC was transfected into SK-N-SH and SK-N-AS cells. As suggested by WB analysis, the HMGB3 protein level was markedly reduced in sh-HMGB3 cells compared to sh-NC cells (*p* < 0.01, Figure 2A). In addition, a CCK-8 assay was conducted to determine the biological effect of HMGB3 on cell proliferation. The results showed that knockdown of HMGB3 remarkably inhibited the growth of NB cells *in vitro* (SK-N-SH, *p* < 0.05; SK-N-AS, *p* < 0.05, Figure 2B).

Similarly, colony formation ability was suppressed in the HMGB3-depleting groups compared to the control group (SK-N-SH, *p* < 0.01; SK-N-AS, *p* < 0.01, Figure 2C). According to the results of the 3D cell culture assay, cell growth was suppressed in the HMGB3-depleted groups compared to the control group (Figure 2D). Wound healing assays indicated that cell migration was inhibited in the HMGB3-silenced groups compared to the control group (SK-N-SH, *p* < 0.01; SK-N-AS, *p* < 0.05, Figure 2E).

**FIGURE 3:**
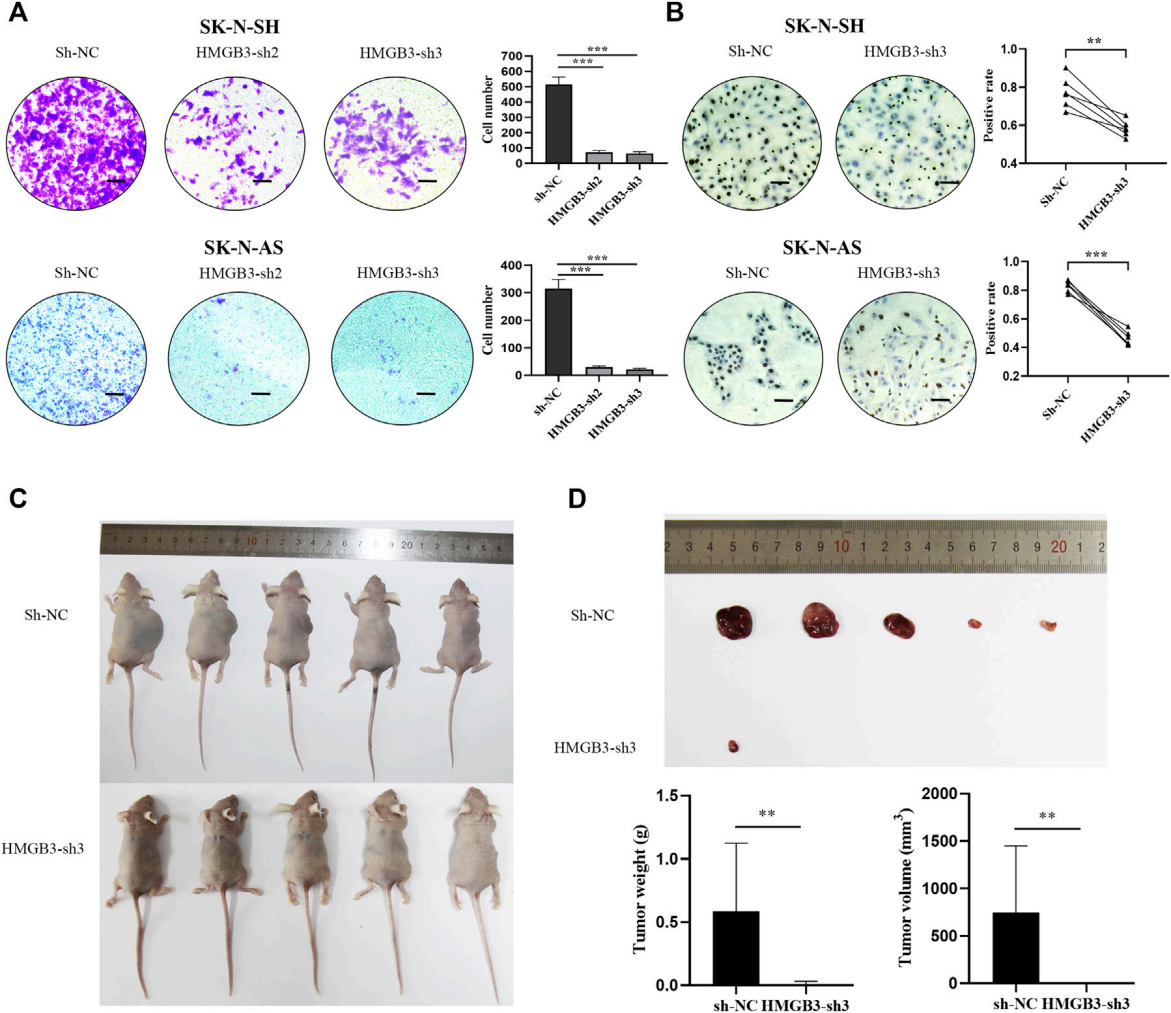
Knockdown of HMGB3 inhibited cell invasion and growth in NB cells *in vitro* and *in vivo*. **(A)** Transwell assay for SK-N-SH and SK-N-AS cells with/without HMGB3 knockdown. **(B)** Immunocytochemistry analysis of KI-67 protein was performed in SK-N-SH and SK-N-AS cells. **(C)** Paired xenograft tumors and their relative injected subcutaneously with sh-NC or sh-HMGB3 SK-N-SH cells. **(D)** Tumor weight. Scale bars, 50 μm *p < 0.05, **p < 0.01, and ***p < 0.001.

Furthermore, the Transwell assay demonstrated that silencing HMGB3 decreased the invasion of NB cells (SK-N-SH, *p* < 0.001, SK-N-AS, *p* < 0.001, Figure 3A). In addition, according to the immunocytochemical analysis, Ki-67 nuclear expression was downregulated in the HMGB3-depleted NB cell lines SK-N-SH (*n* = 6, *p* < 0.01) and SK-N-AS (*n* = 6, *p* < 0.001) compared to the control groups (Figure 3B), indicating that NB proliferation was suppressed upon HMGB3 knockdown. Taken together, these data indicated that HMGB3 plays a critical role in neuroblastoma tumorigenesis.

To further evaluate the function of HMGB3 *in vivo*, we determined whether silencing HMGB3 could inhibit tumor xenograft growth in nude mice. We found that knockdown of HMGB3 did indeed inhibit tumor growth, leading to significantly reduced tumor volume and weight (Figures 3C,D, *p* < 0.01). Thus, our data suggested that silencing HMGB3 inhibits tumor growth *in vivo*.

### HMGB3 Coexpression Genes are Primarily Enriched in Cell Cycle-Related Pathways

**FIGURE 4:**
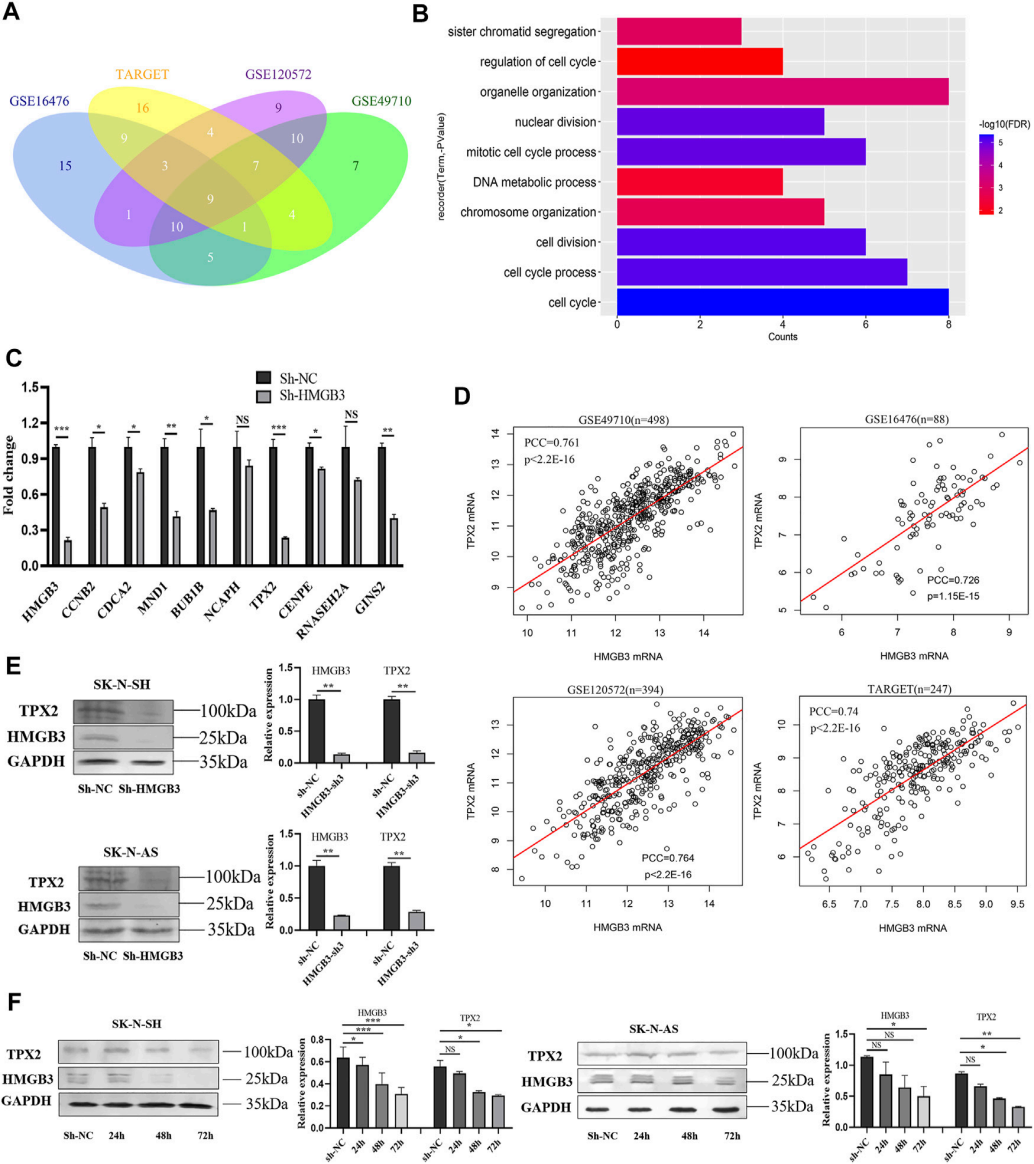
HMGB3 could inhibit cell survival via TPX2 in NB cells. **(A)** Nine genes significantly co-expressed with HMGB3 in four datasets of NB. **(B)** Functional enrichment analysis of nine genes. **(C)** The mRNA expression of nine genes was detected by qRT-PCR in SK-N-SH cells after HMGB3 knockdown. **(D)** Pearson correlation of HMGB3 and TPX2 in four NB datasets. **(E)** Protein expression of TPX2 and HMGB3 was detected by western blotting after HMGB3 knockdown. **(F)** TPX2 reduction was time-dependent with HMGB3 knockdown in SK-N-SH and SK-N-AS cells. *p < 0.05, **p < 0.01, and ***p < 0.001.

In the four NB datasets GSE49710, GSE16476, GSE120572, and TARGET-NBL, genes significantly coexpressed (Pearson correlation coefficient, PCC >0.7) with HMGB3 were selected, among which, nine genes were screened (Figure 4A). According to the functional enrichment analysis of Gene Ontology (GO; biological process, BP), these nine genes were primarily enriched in cell cycle-related pathways, such as regulation of cell cycle, cell cycle process, mitotic cell cycle process, and sister chromatid segregation (Figure 4B). As revealed by univariate Cox regression analysis, these nine genes were risk factors for OS and EFS in NB (Supplementary Figure S2; Supplementary Table S4). Notably, seven of the nine genes were markedly downregulated in SK-N-SH cells upon HMGB3 knockdown, with TPX2 being the most significant (*p* < 0.001, Figure 4C).

On the other hand, the PCCs of TPX2 and HMGB3 were 0.761, 0.726, 0.764, and 0.74 in the above four datasets, respectively (all *p* values <0.001, Figure 4D). Furthermore, the TPX2 protein expression was correspondingly markedly downregulated in NB cells (SK-N-SH, *p* < 0.01; SK-N-AS, *p* < 0.01) upon HMGB3 knockdown (Figure 4E). Specifically, TPX2 expression decreased in a time-dependent manner after HMGB3 knockdown in NB cell lines, which began to decrease at 48 h and was more significant at 72 h (Figure 4F).

### TPX2 Overexpression Reverses the Inhibition of SK-N-SH Cell Proliferation Caused by HMGB3 Knockdown

**FIGURE 5:**
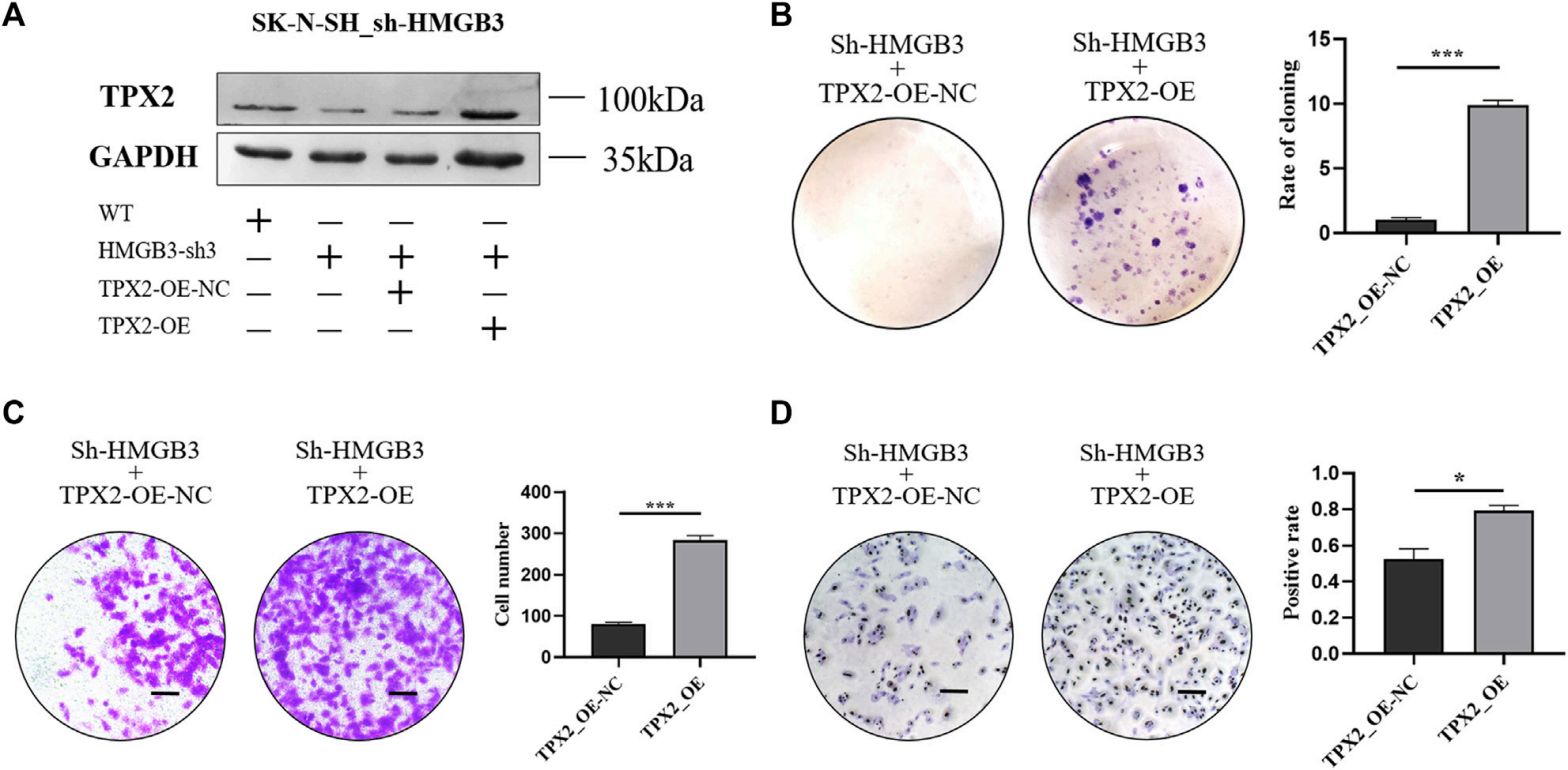
TPX2 overexpression can reverse the inhibition of cell proliferation caused by HMGB3 knockdown in SK-N-SH cells. **(A)** Protein expression was detected in sh-HMGB3 SK-N-SH cells after TPX2 over-expression by western blotting. **(B–D)** Colon formation, Transwell assay, and immunocytochemistry assay for sh-HMGB3 SK-N-SH cells after TPX2 over-expression. Scale bars, 50 μm *p < 0.05, **p < 0.01, and ***p < 0.001.

To investigate the role of TPX2 protein in the HMGB3-mediated promotion of cell proliferation, TPX2 was overexpressed in HMGB3-silenced SK-N-SH cells (Figure 5A). The colony formation ability was reversed in the TPX2-overexpressing groups compared to the control group (*p* < 0.001, Figure 5B). At the same time, the results of the Transwell assay showed that the invasion ability of SK-N-SH cells recovered upon the overexpression of TPX2 (*p* < 0.001, Figure 5C). The number of Ki-67-positive cells also markedly increased in the TPX2 overexpression group compared to the control group (*p* < 0.05, Figure 5D).

### Validation of Prognosis Prediction Performance of HMGB3 and TPX2 in Other Independent NB Datasets

**FIGURE 6:**
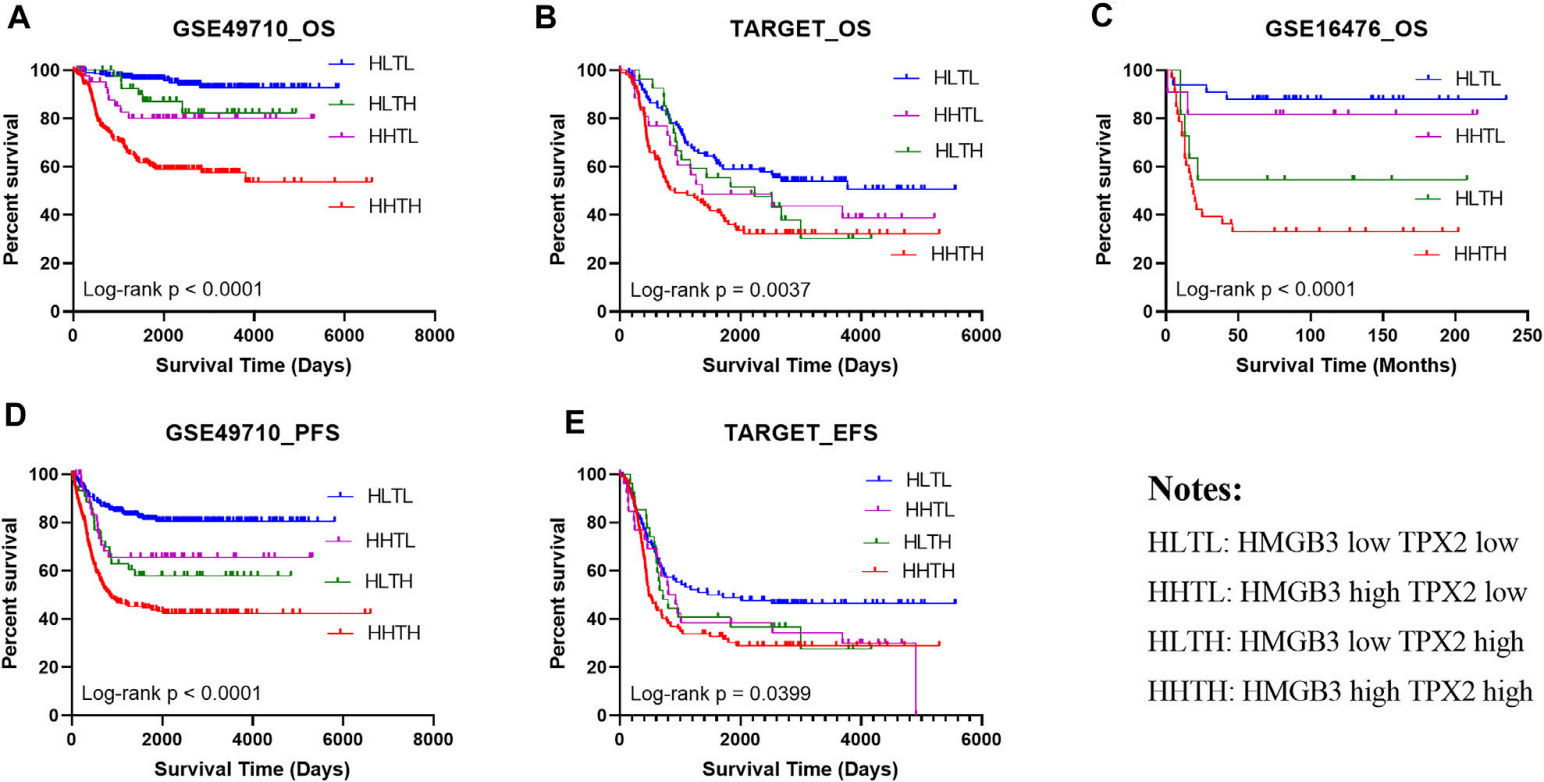
High expression of HMGB3 and TPX2 correlated to inferior NB prognosis in multiple datasets. **(A–C)**, Overall survival (OS) of NB patients in three independent datasets. **(D, E)** Progression-free survival (PFS) and Event-free survival (EFS) in NB patients.

To confirm the prognostic prediction performance of HMGB3 and TPX2, we analyzed the survival of patients with high/low HMGB3 and TPX2 expression in three datasets GSE49710 (*n* = 493), GSE16476 (*n* = 88), and TARGET-NBL (*n* = 247). All patients were classified into four groups based on the median expression level, including HMGB3 high and TPX2 high (HHTH), HMGB3 high, and TPX2 low (HHTL), HMGB3 low and TPX2 high (HLTH), and HMGB3 low and TPX2 low (HLTL). The results showed that patients in the HHTH group exhibited the worst OS, EFS, and PFS, while the HLTL group displayed the most favorable survival (Figure 6), demonstrating that the effects of HMGB3 and TPX2 on survival were superimposed.

**FIGURE 7:**
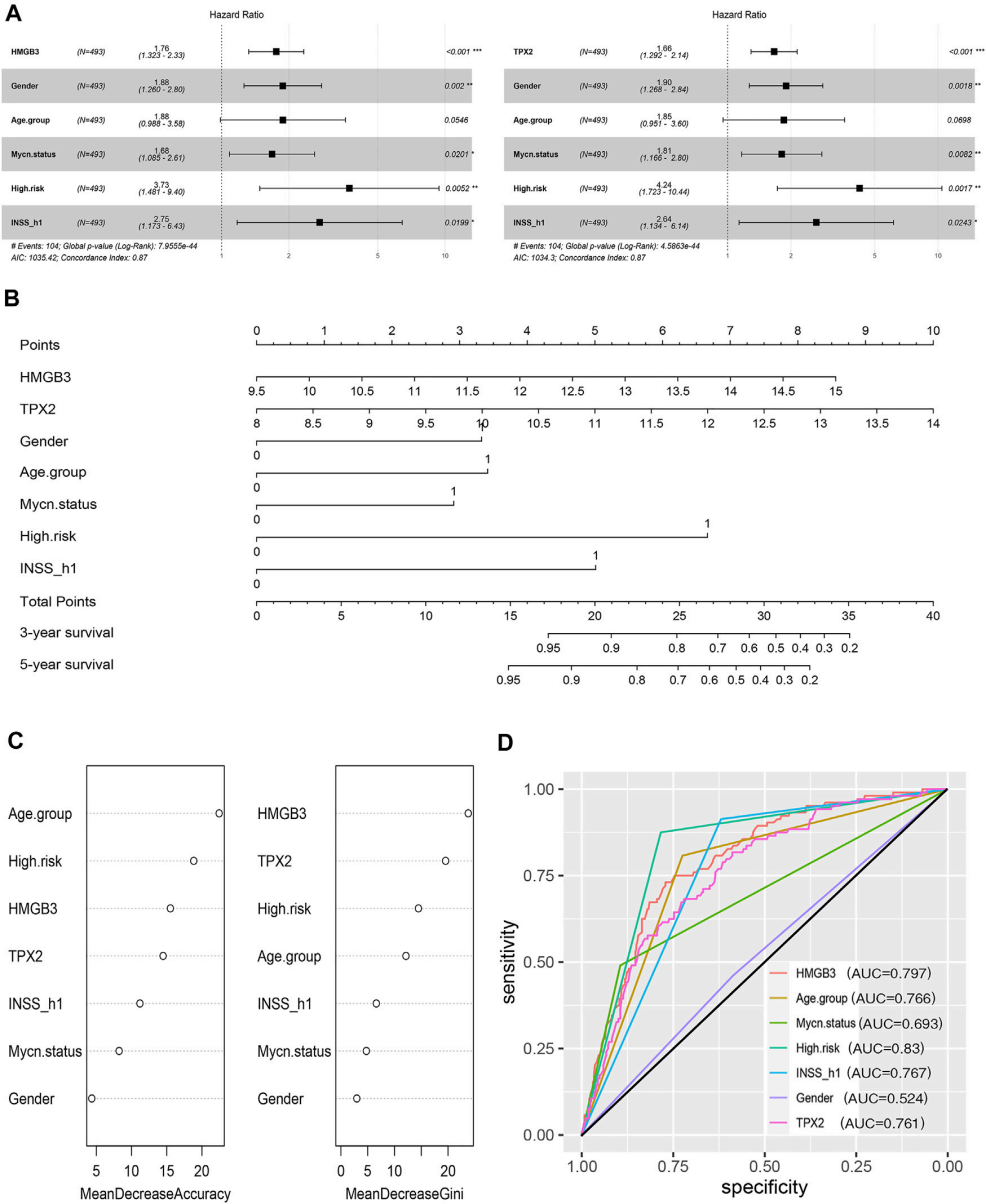
HMGB3 and TPX2 are closely associated with the survival of patients with NB in GSE49710. **(A)** HMGB3 and TPX2 can be independent prognostic factors for overall survival. **(B–D)** Nomogram, random forest, and ROC curves of HMGB3, TPX2 and clinicopathological characteristics for NB patients.

Thereafter, factors including sex, age group (age <18 months vs. age≥ 18 months), MYCN status, high risk, INSS_h1 (INSS stage 1, 2, 4s vs. stage 3, 4), and HMGB3/TPX2 were incorporated for multivariate Cox regression analysis. As a result, HMGB3 and TPX2 might serve as independent prognostic factors for OS and EFS (Figure 7A, Supplementary Figure S1), and had the most significant *p*-values compared to other clinicopathological features. Additionally, a nomogram predictor was constructed based on the expression levels of the two genes and other prognostic features of NB (Figure 7B). The integration of gene expression and clinicopathological features exhibited better predictive power for prognosis.

To estimate the importance of HMGB3 and TPX2 in determining the vital status of NB patients, we employed the machine learning algorithm-random forest. Typically, MDA, and MDG are the parameters used to evaluate the importance (Wang et al., 2016). According to the ranking of importance by the random forest algorithm, TPX2 and HMGB3 occupied more important positions than the other features (Figure 7C). At the same time, we used ROC curve analysis to assess the prediction abilities of the two genes and other risk factors. The results showed that when predicting the vital status of patients, the AUC values ranging from highest to lowest were high risk, HMGB3 expression, INSS_h1, age, TPX2 expression, and MYCN status (Figure 7D). Both HMGB3 and TPX2 expression displayed good predictive performance, with AUC values of 0.797 and 0.761, respectively.

## Discussion

NB is a highly heterogeneous tumor, and the long-term survival for high-risk patients remains poor and is still below 50% despite aggressive multimodal treatment (Pugh et al., 2013; Wienke et al., 2021). Treatment for high-risk or refractory/relapsed NB has shifted to a combination of classical treatment (chemotherapy, surgical treatment, radiotherapy, and stem cell transplantation) with targeted drug therapy or immunotherapy (Matthay, 2018). However, identifying new targets remains challenging.

The HMGB family plays an important role in many cancers. In this article, we analyzed the expression of HMGB1, HMGB2, and HMGB3 in different prognostic groups of NB patients. Our results suggested that HMGB3 exhibited the most significant difference and was highly expressed in patients with unfavorable prognoses. Dysregulation of the WNT signaling pathways in carcinogenesis is observed in multiple solid and liquid tumors (Zhan et al., 2017). Studies have indicated that HMGB3 promotes cancer cell proliferation by activating the WNT/β-catenin pathway (Zhang et al., 2017; Xie et al., 2019; Li Y. et al., 2020; Zhuang et al., 2020). Gu et al. (2019) discovered that HMGB3 silencing inhibited BC growth by interacting with HIF-1α. In addition, HMGB3 is correlated with treatment resistance. Li Z et al. (2020b) demonstrated that HMGB3 enhanced radioresistance by binding to the promoter region of hTERT in cervical cancer and suggested that targeting the HMGB3/hTERT axis might help cervical cancer patients who suffer from radioresistance. In ovarian cancer, Mukherjee et al. (2019) suggested that HMGB3 depletion might sensitize chemoresistant cancer cells to cisplatin through the ATR/CHK1/p-CHK1 DNA damage signaling pathway.

Based on the data mining results, we found that HMGB3 expression was increased in NB patients with unfavorable prognosis, and its high expression predicted inferior survival. According to data analysis and evidence from the literature, we speculated that HMGB3 plays an oncogenic role in NB progression. We further confirmed our assumption using a loss-of-function test. Specifically, we silenced HMGB3 expression in NB cell lines and detected the survival of NB cells. The results revealed that cell proliferation, migration, and invasion were inhibited. *In vivo*, HMGB3 knockdown inhibited NB tumor development in mice. Simultaneously, Ki-67 expression decreased upon HMGB3 knockdown. These results suggested that HMGB3 is essential for cell survival and biological function in NB progression.

By coexpression analysis, nine genes coexpressed with HMGB3 were selected, and their high expression levels predicted inferior survival. Moreover, functional enrichment analysis (Consortium, 2016) demonstrated that these genes were primarily enriched in cell cycle-related pathways. The above results demonstrated that the cell cycle played an important role in the survival of NB cells and the prognosis of NB patients.

Subsequently, we detected the mRNA expression levels of these nine genes in sh-HMGB3 cells, among which, seven were markedly downregulated after HMGB depletion, including CCNB2, CDCA2, MND1, BUB1B, TPX2, CENPE, and GINS2. CCNB2 is highly expressed in lung adenocarcinoma (LUAD) (Wang et al., 2020) and hepatocellular carcinoma (HCC) (Li et al., 2019), and is correlated with poor prognosis. CDCA2 promotes cancer cell proliferation in melanoma (W.-H. JIN et al., 2020) and colorectal cancer (CRC) (Feng et al., 2019). MND1 regulates cell cycle progression by forming a feedback loop with KLF6 and E2F1 in LUAD both *in vitro* and *in vivo* (Zhang et al., 2021). Furthermore, the high expression of BUB1B is associated with adverse clinicopathological characteristics of HCC, which plays an oncogenic role by upregulating the mTORC1 signaling pathway (Qiu et al., 2020). CENPE is highly expressed in LUAD specimens and promotes cancer cell proliferation regulated by FOXM1 (Shan et al., 2019). CINS2 promotes epithelial-mesenchymal-transition (EMT) in pancreatic cancer by activating the ERK/MAPK signaling pathway (Huang et al., 2020). GINS2 silencing inhibits (Sun et al., 2021) cell proliferation, growth, and cell cycle arrest at the G2/M phase *in vitro* and *in vivo* by suppressing the STAT signaling pathway (Huang et al., 2020). Several studies have demonstrated that TPX2 is correlated with the response to DNA damage (Neumayer et al., 2014; Neumayer and Nguyen, 2014). Ognibene M. (Ognibene et al., 2019). found that increased expression of the TPX2 oncoprotein repaired DNA damage in NB, which predicted poor prognosis of NB patients.

Seven of the genes coexpressed and changed with HMGB3 exhibit carcinogenic effects in various tumors. Of the seven genes, TPX2 expression displayed the most significant decrease. At the protein level, TPX2 expression was also reduced after HMGB3 knockdown in a time-dependent manner. The correlations of TPX2 with the clinicopathological features of NB were consistent in our study. We then constructed a gain-of-function model, and the results showed that overexpression of TPX2 partially relieved the inhibitory effect of HMGB3 silencing.

HMG family proteins can regulate transcription and modify chromatin structure by binding DNA in a structure-dependent manner, so they are figuratively known as “architectural transcription factors”. HMGB1 possesses the A and B box domains, which can bind to noncanonical DNA structures and damaged DNA to affect DNA damage and repair and play intracellular roles (Lange and Vasquez, 2009). HMGB1 is an important paralog of HMGB3, hence, they have similar structures and functions. Taken together, we speculated that HMGB3 might act as a transcriptional regulatory switch to regulate the expression of a series of genes by binding to target DNA in the nucleus, and this conjecture was preliminarily confirmed with TPX2.

According to an ancient Chinese saying, “Destroy the leader and the gang will collapse”. Our results suggested that inhibiting HMGB3 inhibits several related oncogenes, and HMGB3 might represent an ideal therapeutic target for NB. Our future studies will explore the binding sites of targeted DNAs to further understand the role of HMGB3 in regulating multiple genes in NB in the future.

In conclusion, based on data mining and biological experiments, our studies identify that HMGB3 plays an oncogenic role by regulating TPX2 in NB. The effects of HMGB3 on NB not only provide new insight into the survival mechanism of cancer cells but also reveal a potential implication of HMGB3 in prognosis and a novel therapeutic strategy for NB.

## Data Availability

Publicly available datasets were analyzed in this study. This data can be found here: GSE49710, GSE16476, and GSE120572 (https://www.ncbi.nlm.nih.gov/gds/?term=) and TARGET-NBL (https://hgserver1.amc.nl/cgi-bin/r2/main.cgi).
