# Supplementary material for: HMGB3 is Associated With an Unfavorable Prognosis of Neuroblastoma and Promotes Tumor Progression by Mediating TPX2
Source: Front Cell Dev Biol. 2021 Dec 20;9:769547. doi: 10.3389/fcell.2021.769547 (PMC8721485; doi:10.3389/fcell.2021.769547)
Supplement: Supplementary file 1 [file DataSheet1.pdf]

Supplementary Material

1 Supplementary Figures

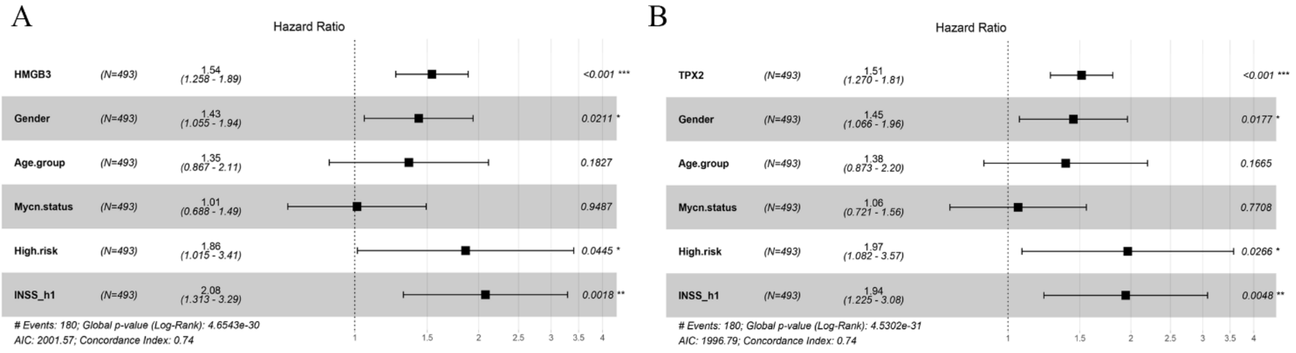

Supplementary Figure 1. HMGB3 and TPX2 can be independent prognostic factors for progression-free survival. (**A)** ggforest for HMGB3 and clinicopathological features. (**B)** ggforest for TPX2 and clinicopathological features. *p<0.05, **p<0.01 and ***p<0.001

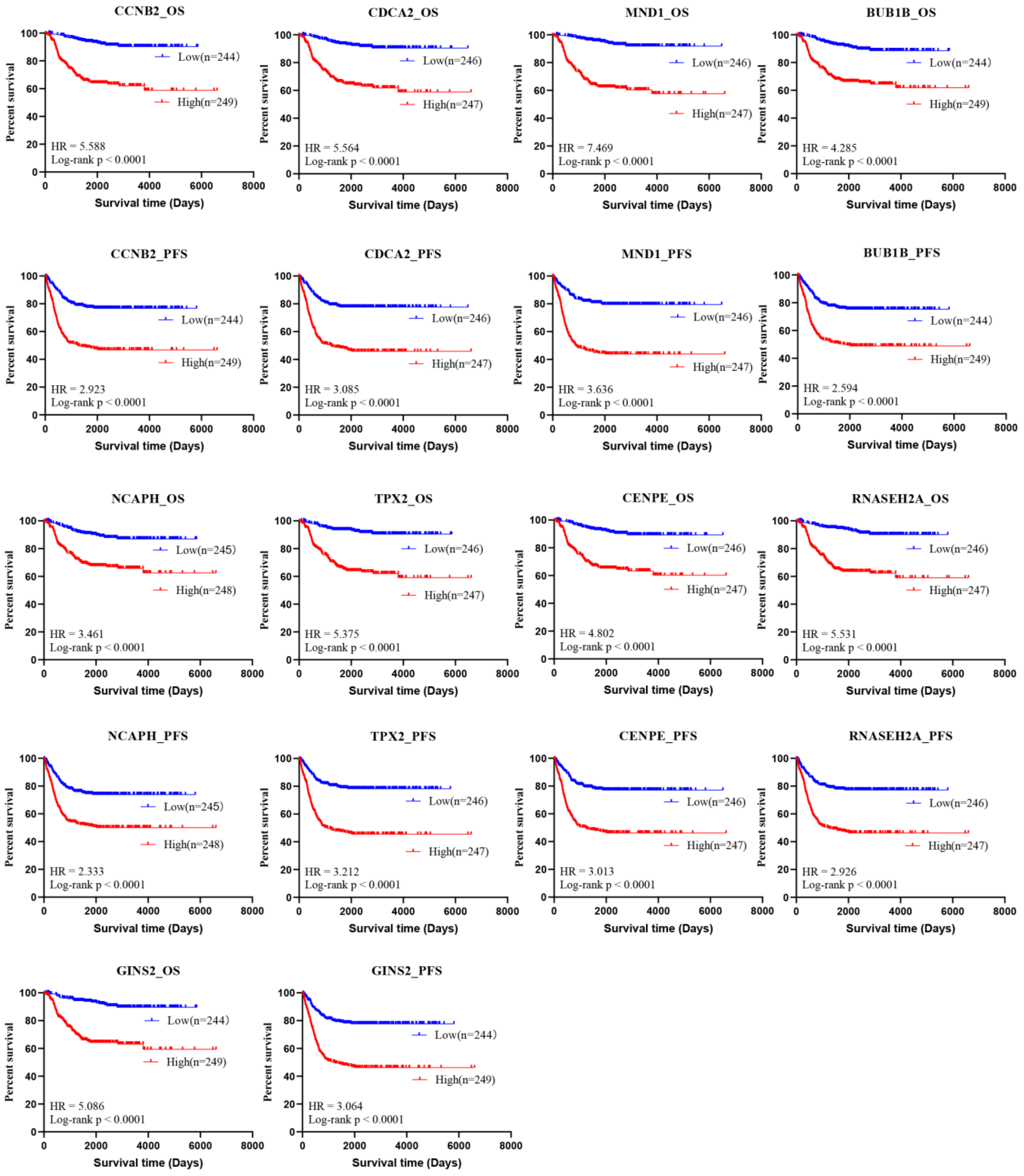

SUPPLEMENTARY Figure 2. Nine genes co-expressed with HMGB3 are associated with the survival of patients in GSE49710. OS, overall survival; PFS, progression-free survival.

2 Supplementary Tables

Supplementary Table 1. The relationship between HMGB3 expression and clinicopathological features in NB patients in GSE16476.

**Features**	**HMGB3 Expression**			
	**High**	**Low**	**χ2**	**p value**
**Gender**				
Male	27	26	0	1
Female	17	18		
**Age group**				
≥18 months	28	12	10.312	0.001
<18 months	16	32		
**MYCN status**				
Amplified	11	5	1.91	0.167
Non-amplified	33	39		
**Stage**				
3, 4	29	11	13.246	2.73E-04
1, 2, 4s	15	33		
**Recurrence**				
Yes	25	9	10.784	0.001
No	19	35		
**Death from disease**				
Yes	24	9	9.503	0.002
No	20	35		

Supplementary Table 2. The relationship between HMGB3 expression and clinicopathological features in NB patients in TARGET-NBL.

Features	HMGB3 Expression			
	High	Low	χ2	p value
**Gender**				
Male	65	77	2.22	0.136
Female	59	46		
**Age group**				
≥18 months	118	97	13.138	2.89E-04
<18 months	6	26		
**MYCN status**				
Amplified	54	14	30.615	3.15E-08
Non-amplified	68	107		
**High risk**				
Yes	122	95	23.945	9.91E-07
No	2	28		
**Stage**				
3, 4	122	95	23.945	9.91E-07
1, 2, 4s	2	28		
**Event**				
Yes	87	69	4.662	0.031
No	37	54		
**Death**				
Yes	79	61	4.453	0.035
No	45	62		
**Ploidy**				
Diploid (DI=1)	40	23	6.221	0.013
Hyperdiploid (DI>1)	44	60		
**Histology**				
Unfavorable	106	77	25.473	4.49E-07
Favorable	4	33		
**Grade**				
Undifferentiated or Poorly Differentiated	90	80	5.394	0.02
Differentiating	2	11		
**MKI**				
High	41	11	22.955	1.66E-06
Low/Intermediate	47	77		

Supplementary Table 3. The relationship between HMGB3 expression and clinicopathological features in NB patients in GSE120572.

Features	HMGB3 Expression			
	**High**	**Low**	**$\chi 2$**	**p value**
**Age group**				
⩾18 months	66	48	5.61	0.018
<18 months	38	56		
**MYCN status**				
Amplified	44	8	31.915	1.61E-08
Non-amplified	59	96		
**Stage**				
3, 4	73	45	14.278	1.58E-04
1, 2, 4s	31	59		
**Event**				
Yes	49	33	4.53	0.033
No	55	71		
**TERT rearrangement**				
Positive	12	9	0.212	0.645
Negative	92	95		

Supplementary Table 4. The genes co-expressed with HMGB3.

Gene Symbol	GSE16476	GSE49710	TARGET-249	GSE120572	Chr	HR	95%CI	p. value	C-index	AUC_vital status
HMGB3	1	1	1	1	Xq28	3.138	2.492-3.95	<2E-16	0.787	0.797
CCNB2	0.767	0.77	0.733	0.773	15q22.2	2.36	1.959-2.842	<2E-16	0.783	0.784
CDCA2	0.764	0.759	0.721	0.766	8p21.2	2.487	2.055-3.011	<2E-16	0.78	0.787
MND1	0.716	0.772	0.746	0.774	4q31.3	2.68	2.232-3.218	<2E-16	0.807	0.816
BUB1B	0.748	0.752	0.756	0.787	15q15.1	2.396	1.931-2.973	2.02E-15	0.745	0.755
NCAPH	0.727	0.763	0.727	0.784	2q11.2	2.203	1.796-2.707	3.44E-14	0.738	0.745
TPX2	0.726	0.761	0.739	0.764	20q11.21	2.521	2.022-3.144	<2E-16	0.753	0.761
CENPE	0.725	0.778	0.742	0.789	4q24	2.58	2.111-3.153	<2E-16	0.77	0.778
RNASEH2A	0.721	0.806	0.739	0.799	19p13.13	3.209	2.503-4.114	<2E-16	0.769	0.777
GINS2	0.721	0.789	0.767	0.789	16q24.1	2.146	1.77-2.602	8.02E-15	0.74	0.748

Notes: Chr, chromosome; HR, hazard ratio; CI, confidence interval.
